# Characterization of the Juvenile Hormone Pathway in the Viviparous Cockroach, *Diploptera punctata*

**DOI:** 10.1371/journal.pone.0117291

**Published:** 2015-02-23

**Authors:** Juan Huang, Elisabeth Marchal, Ekaterina F. Hult, Stephen S. Tobe

**Affiliations:** 1 Department of Cell and Systems Biology, University of Toronto, Toronto, Canada; 2 Department of Biology, Zoological Institute, KU Leuven, Leuven, Belgium; University of Freiburg, GERMANY

## Abstract

Juvenile hormones (JHs) are key regulators of insect development and reproduction. The JH biosynthetic pathway is known to involve 13 discrete enzymatic steps. In the present study, we have characterized the JH biosynthetic pathway in the cockroach *Diploptera punctata*. The effect of exogenous JH precursors on JH biosynthesis was also determined. Based on sequence similarity, orthologs for the genes directly involved in the pathway were cloned, and their spatial and temporal transcript profiles were determined. The effect of shutting down the JH pathway in adult female cockroaches was studied by knocking down genes encoding HMG-CoA reductase (HMGR) and Juvenile hormone acid methyltransferase (JHAMT). As a result, oocyte development slowed as a consequence of reduction in JH biosynthesis. Oocyte length, fat body transcription of *Vg* and ovarian vitellin content significantly decreased. In addition, silencing *HMGR* and *JHAMT* resulted in a decrease in the transcript levels of other genes in the pathway.

## Introduction

Juvenile hormones (JHs) play key roles in regulating growth, development, metamorphosis, aging, caste differentiation and reproduction in insects (as reviewed by [1,2,3]). The multiple processes in which JH is involved and the critical role which JH plays in metamorphosis and reproduction emphasize the importance of elucidating the JH biosynthetic pathway and the factors that regulate its biosynthesis.

**Fig 1:**
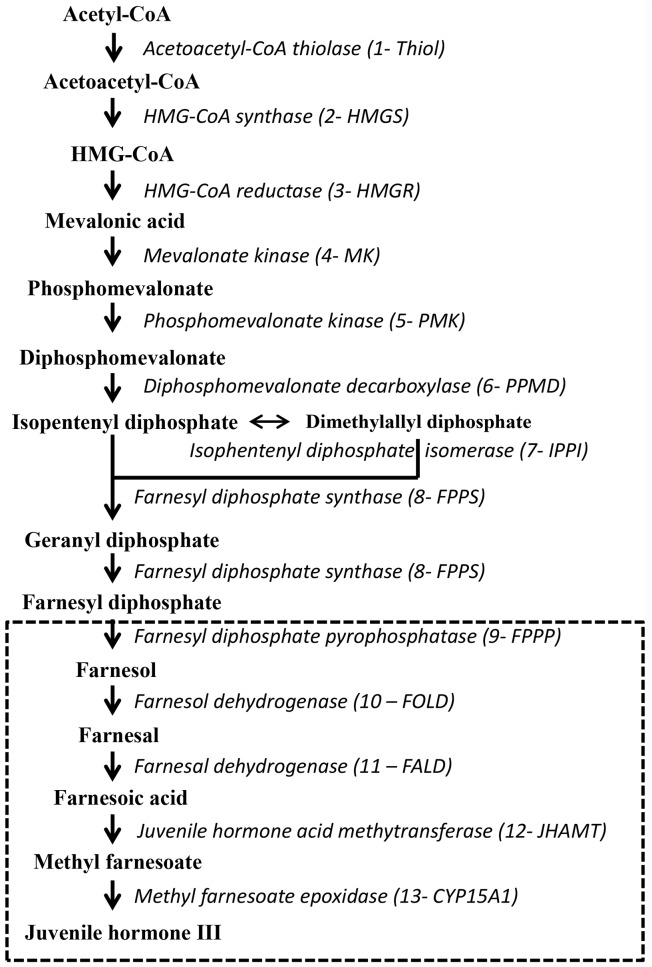
Scheme of JH biosynthetic pathway (Adapted from Belles et al. [4] and Nouzova et al. [11]). The Insect- and Arthropod-specific isoprenoid branch of JH biosynthesis is represented in the dashed box. Precursors are in bold and connected by arrows. Enzymes are in italics. Abbreviations for the enzymes are given in brackets.

JHs are sesquiterpenoid compounds that are synthesized and secreted by specialized, paired endocrine glands, the corpora allata (CA). The complete biosynthetic pathway of JH III (the most widespread and predominant JH homolog in insects) comprises 13 discrete enzymatic steps. This pathway can be divided into two metabolic parts (Fig. 1): the early portion comprises the mevalonate pathway to the formation of farnesyl diphosphate (FPP) and is conserved in both vertebrates and invertebrates; the later part of the pathway is specific to insects and other arthropods. In this later part, FPP is cleaved to farnesol, which is then oxidized to the carboxylic acid (farnesoic acid; FA), followed by methyl esterification, epoxidation and formation of JH [4]. The order in which these two final steps in JH biosynthesis occurs, appears to be insect order dependent. In orthopteran, coleopteran, dipteran and dictyopteran insects, FA is first methylated to methyl farnesoate (MF), which in turn undergoes a C10, C11 epoxidation to the functional JH. In Lepidoptera, however, the opposite situation prevails: epoxidation precedes methylation [5–7].

Recent studies have reported on the molecular elucidation of the JH pathway in several holometabolous insects such as the silkworm *Bombyx mori*, the mosquito, *Aedes aegypti* and the honeybee, *Apis mellifera*. In *B*. *mori*, all genes encoding enzymes involved in the mevalonate pathway [8] and the isoprenoid branch of JH biosynthesis [7,9] have been isolated. Each enzyme in the mevalonate pathway is encoded by a single gene, except farnesyl diphosphate synthase (FPPS), which comprises three homologs. The genes encoding enzymes in the isoprenoid branch of JH biosynthesis, however, underwent gene duplication to create multiple copies. The transcripts for most JH enzymes are highly enriched or exclusively expressed in the CA [10]. The expression pattern of the genes encoding these enzymes in the CA correlates well with rates of JH biosynthesis [8]. In *A*. *aegypti*, changes in the transcription of 11 of the enzymes are responsible in part for the dynamic changes in JH biosynthesis [11]. The expression of genes in the JH biosynthetic pathway was also determined in female castes of *A*. *mellifera* and was found to correlate with the JH hemolymph titre in adult worker bees, but, not in larvae [12]. Until recently, no structural or molecular data were available on FPP pyrophosphatase (FPPP) or farnesal dehydrogenase (FALD). Current studies in the dipterans, the fly, *Drosophila melanogaster* and *A*. *aegypti* have characterized the gene encoding an FPP phosphatase (FPPP) [13,14]. Moreover, genes encoding an aldehyde dehydrogenase (FALD) have now been functionally characterized in *A*. *aegypti* [15].

*Diploptera punctata*, the only truly viviparous cockroach, is a well-known model system in the study of JH biosynthesis and its regulation. The physiology of this animal is characterized by very stable and high rates of JH biosynthesis and precise and predictable reproductive events that correlate well with rates of JH production (see review by Marchal et al. [16]). In adult females, JH regulates oocyte maturation, fat body vitellogenin (Vg) production and the uptake of Vg by the developing oocytes [17,18]. Aside from the original molecular identification of *CYP15A1* [19], the gene encoding the epoxidase producing the functional JH, JH-related research in *D*. *punctata* has mainly focused on examining JH titre and physiological aspects of JH function (Marchal et al. [16]).

An earlier study predicted 4 genes encoding enzymes in the JH biosynthetic pathway in *D*. *punctata* based on sequence similarity with *Drosophila* and *Anopheles gambiae* genes [20]. To further characterize the JH biosynthetic pathway in this important model system on a molecular level, we have confirmed and identified 11 out of 13 genes encoding the JH biosynthetic enzymes, and have also analyzed the tissue distribution and developmental transcript profile of these genes during the first gonadotropic cycle of the female cockroach. Our realtime profiling data suggest that the 11 genes cloned in our study are encoding functional enzymes. Moreover, our results suggest that the rate of JH biosynthesis is regulated by the flux of substrates in the pathway as well as by expression of genes in the JH biosynthetic pathway. In a follow-up RNA interference (RNAi) study, we examined the effect of silencing *HMGR* and *JHAMT* on rates of JH biosynthesis and ovarian development. By directly blocking JH biosynthesis, the quantity of Vg mRNA in the fat body significantly decreased and subsequently ovarian development slowed. Of particular interest is our discovery that manipulation of individual genes encoding the JH biosynthetic enzymes using dsRNA technology resulted in a significant decrease in the transcript levels of other genes, which indicates a feedback mechanism is involved in the regulation of the expression of enzymes in the JH biosynthetic pathway.

## Materials and Methods

### Animals

The *D*. *punctata* colony was maintained at 27°C in constant darkness and animals were fed lab chow and water *ad libitum*. To obtain pools of synchronised animals, newly molted female adult cockroaches were picked from the colony, placed in separate containers and provided with water and lab chow. Mated status was confirmed by the presence of a spermatophore.

### Tissue collection and RNA extraction

*D*. *punctata* were dissected in modified cockroach ringer solution (150 mM NaCl, 12 mM KCl, 10 mM CaCl_2_.2H_2_O, 3 mM MgCl_2_.6H_2_O, 10 mM HEPES, 40 mM glucose, pH 7.2) using a dissecting microscope. Samples were flash-frozen in liquid N_2_ to prevent RNA degradation and were stored at −80°C until further processing. For each dissected female, basal oocyte lengths were measured to determine the physiological age. CA samples were taken from day 0 to 7 of adult females for the developmental profiles of genes encoding enzymes in the JH biosynthetic pathway. For each time point, three biologically independent pools of 10 animals each were collected. To determine the tissue distribution of the genes of interest, the following tissues were dissected from 3 independent pools of 10 animals each: brain (Br), nerve cord (NC), corpora allata (CA), fat body (FB), midgut (MG), Malpighian tubules (MT), ovary (Ov) from females, and accessory gland (AG) and testes (Te) from males. For the RNAi experiments (§2.6), 3 biologically independent pools comprising CA from 7 animals were collected. Pooled samples were homogenised with RNase-free pestles and total RNA was extracted using the RNeasy Mini Kit (Qiagen) according to the manufacturer’s instructions. An additional DNase treatment (RNase-free DNase set, Qiagen) was performed to eliminate potential genomic DNA contamination. Because of the small size of the CA, RNA from this tissue was extracted using the RNAqueous-Micro Kit (Ambion), followed by the recommended DNase step. Quality and concentration of the resulting RNA samples were measured using a Nanodrop spectrophotometer (Thermo Scientific.).

### Sequencing genes involved in the JH biosynthetic pathway

Since no genome or full transcriptome sequence data are currently available for *D*. *punctata*, a first set of degenerate primers was developed based on a multiple sequence alignment of known orthologous sequences from different insects. Such an alignment was made for Acetoacetyl-CoA thiolase (Thiol), HMG-CoA reductase (HMGR), Phosphomevalonate kinase (PK), Diphosphomevalonate decarboxylase (PPMD), Isopentenyl diphosphate isomerase (IPPI), Juvenile hormone acid methyltransferase (JHAMT) and the JH target vitellogenin (Vg). Degenerate primers are listed in S1 Table. For Methyl farnesoate epoxidase (CYP15A1) the full sequence was already characterised (GenBank accession number AY509244) [19]. For HMG-CoA synthase (HMGS), Mevalonate kinase (MK), Farnesyl diphosphate synthase (FPPS) and Farnesol dehydrogenase (FOLD), a partial sequence was found in the *D*. *punctata* CA EST database [20]. Their sequence was partially confirmed using gene-specific primers (S1 Table). A temperature-gradient polymerase chain reaction (PCR) was run using Taq DNA polymerase (Sigma-Aldrich Co.) with *D*. *punctata* day 4 CA cDNA as a template. Bands of the expected size were cut out and further purified using the GenElute Gel extraction Kit (Sigma-Aldrich Co.). The resulting DNA fragments were subcloned into a CloneJET cloning vector using the CloneJET PCR Cloning Kit (Fermentas) and sequenced. Sequences for *PMK*, *PPMD* and *JHAMT* identified using degenerate primers, were too short to submit to NCBI’s GenBank; therefore, RACE (Rapid Amplification of cDNA Ends) was performed. Their sequences were confirmed using primers listed in S1 Table.

### Radiochemical assay (RCA)

The *in vitro* radiochemical assay (RCA) for JH biosynthesis was performed [21,22]. CA were incubated in TC199 medium for 3h, then transferred to new medium containing JH precursors for another 3h incubation. JH biosynthesis in both incubations was measured, and first incubation measurements were used as a control. JH precursors acetyl CoA, mevalonic acid (MA), diphosphomevalonate (DPPM) and farnesol (Sigma-Aldrich, Canada) were dissolved in water before use.

### cDNA synthesis and quantitative real-time PCR (q-RT-PCR)

cDNA was transcribed from an equal amount of RNA using the SuperScript III First-Strand Synthesis SuperMix for q-RT-PCR in a final volume of 20 μl following the manufacturer’s instructions (Invitrogen Life Technologies). All samples were reverse transcribed together in a single run. The resulting cDNA samples were diluted 10-fold with PCR grade water. A calibrator sample was prepared by pooling 5 μl of each cDNA sample. In the same run, negative control reactions were set up without reverse transcriptase enzyme to test for genomic DNA contamination.

q-RT-PCR primers were designed using IDT’s (Integrated DNA Technologies) PrimeQuest design tool (http://eu.idtdna.com/PrimerQuest/Home/Index). Primer sets were subsequently validated by determining relative standard curves for each gene transcript using a five-fold serial dilution of the calibrator cDNA sample. Efficiency and correlation coefficients (R^2^) were determined for each primer pair. Primers used for q-RT-PCR profiling are listed in Table 1.

**Table 1:** q-RT-PCR primer sequences and reaction efficiencies and correlation coefficients in the q-RT-PCR assay.

Gene name	F primers	R primers	Efficiency (%)	Correlation coefficient (R^2^)
***Thiol***	5′-TGCCTTCCAAAAGGAGAATG-3′	5′- ACATCACCTGCCATCAACAC-3′	90	0.97
***HMGS***	5′- TGCTGGGAAGTACACAGCAGG-3′	5′- CTCCACGAGCTTGCTGACTG-3′	83	0.994
***HMGR***	5′-TGGGAGCATGTTGTGAAAAT-3′	5′-ACCAAGCAGCCCTCAGTAGT-3′	95	0.988
***MK***	5′- TACGGCAAAACTGCCCTTGC-3′	5′- AATGGAGGAGGTTCGGCGA-3′	93	0.996
***PMK***	5′- TACGAAAACAACGAGGATGG-3′	5′- TTCTGCATCATCTACACCTTCA-3′	100	0.985
***PPMD***	5′- TGGAAGGTGACATAACAGCAA-3′	5′- ATCCTTGATGCCAGTGAACA-3′	90	0.967
***IPPI***	5′- CCTTCCCCAACCATGTAACT-3′	5′- ACCAACGCCATTTGTCTCTT-3′	100	0.992
***FPPS***	5′-TGCTTTGGAGATCCTGAGGT-3′	5′- TGTTCAGGAGTGGTTCGTTG-3′	96	0.987
***FOLD***	5′- TGGCGCGTAGGGTAGACAA-3′	5′- GACCCATTTGAAAGCCTCCTTGA-3′	93	0.993
***JHAMT***	5′- ATCCAGGTGCTGGAAGGAGAG-3′	5′- CTGCCCAGAGTCGAACAGG-3′	99	0.984
***CYP15A1***	5′- GTTGGGATCTCGGAGCATGG-3′	5′- CGAACACGTCATGCATCGGT-3′	100	0.992
***Vg***	AAAGGTGTCCTCAGCCAGC	TCCTCCATCTCGGATTGGGA	95	0.998

For reference gene q-RT-PCR specifications, the reader is referred to Marchal et al. [20].

q-RT-PCR reactions were carried out in triplicate in a total volume of 10 μl containing 5 μl of IQ SYBR Green Supermix (Bio-Rad), 1 μl forward and reverse primer (5 μM), 2 μl of MQ-water and 1 μl of cDNA. All reactions were performed using Bio-Rad’s CFX384 Touch Real-Time PCR Detection System using a two-step thermal cycling profile: 95°C for 3 min, followed by 40 cycles of 95°C for 10 s and 59°C for 30 s. Upon completion of every run, a dissociation protocol (melt curve analysis) was performed to check for formation of primer dimers. A few representative PCR products were run on a 1.2% agarose gel containing GelRedTM (Biotium) and visualised under UV to confirm target specificity. Prior to target gene profiling, previously described housekeeping genes were tested for their stability in the designed tissue distribution, temporal profiling and RNAi experiments. The optimal housekeeping genes were selected using the geNorm and Normfinder software as described previously [23]. For each tested cDNA sample, the normalization factor for the reference genes relative to the calibrator samples was calculated and used to determine the normalized expression levels of the target genes relative to the calibrator [24].

### RNA interference (RNAi)

CA cDNA was used as a template to amplify fragments of genes to be used in dsRNA construction. These fragments were subcloned and sequenced as described above. Two separate constructs were designed in different regions of the genes to eliminate off-target effects. Primers used are given in Table 2. dsRNA was synthesized using the MEGAscript RNAi kit (Ambion). The procedure is based on the high-yield transcription reaction of a user-provided linear transcript with a T7 promoter sequence. Transcription was carried out at 37°C overnight. The reaction mixture was treated with DNase I and RNase, and then purified by phase-solid phase adsorption purification, according to the manufacturer’s instructions (Ambion). The dsRNA concentration was determined using a Nanodrop spectrophotometer (Thermo Scientific.). Diluted dsRNA was run on a 1.2% agarose gel to examine integrity of the construct and efficiency of duplex formation. The negative control construct (-pJET) was designed in a non-coding region of the pJET 1.2/blunt cloning vector (CloneJet PCR Cloning Kit, Thermo Scientific).

**Table 2:** Primers for dsRNA construction.

Name	F Primer(5′-3′)	R Primer(5′-3′)
**HMGR dsRNA**	**ACATGGACAGTTCTGTGCCT**	**CCCAACTTTTGCAGATGACAG**
	**CACTTCTCGCATTGTGGCT**	**CAGTACCCTTGGAGAGC**
**JHAMT dsRNA**	**AAAAGAGACGCAGCCCACGCA**	**CGATCCTCGTGGGAACAGATG**
	**GTACAGCACGCCACCTCCA**	**AACTACGGCACTCTGGAGC**
**Control dsRNA**	**TTGCGCTCACTGCCAATTGC**	**CTGGCCTTTTGCTCACATGTT**

*The T7 promoter sequence was added at the 5′ end of each dsRNA primer.

Several RNAi trials with different injection timings and dsRNA construct concentrations were investigated to obtain an efficient gene silencing (data not shown). Based on the results of RNAi trials, newly molted mated females were injected with 3 μg *HMGR* dsRNA on day 0 and day 2, and with 3 μg *JHAMT* dsRNA on day 1 and day 3. CA, ovary and fat body samples were taken on day 4 as described above. Fat body was immediately stored in liquid N_2_ prior to RNA extraction. CA were dissected and cleaned in TC199 medium (GIBCO; 1.3 mM Ca2+, 2% Ficoll, methionine-free) for use in the RCA or flash frozen in liquid N_2_ to prevent RNA degradation. Basal oocyte length was measured and the ovaries were collected for vitellogenin measurements (§ 2.7) and histology (§ 2.8).

### Vitellogenin ELISA

Single dissected ovaries were homogenized and extracted twice in 50 μl PBS according to Mundall et al. [25]. Total ovary protein content was determined using a Bradford assay [26] performed according to the manufacturer’s (Sigma-Aldrich) 96 well plate protocol.

Vitellin was quantified using an indirect enzyme-linked immunosorbent assay (ELISA) following the protocol described in Marchal et al. [27]. A rabbit polyclonal antibody made against *D*. *punctata* mature egg homogenate was used as the first antibody [28]. Goat anti-rabbit IgG, HRP-linked antibody (1:3000 in 1% BSA) was then added as the second antibody. Wells were treated with 100ul of TMB for 10 min. Absorbance was measured at 650 nm with a Molecular Devices SpectraMax Pus 384 microplate reader.

### Histology and microscopy

To view overall structure, a subset of oocytes was fixed for 3 days in 3% glutaraldehyde in 0.1 M phosphate buffer, post-fixed for 1h with 1% OsO_4_ in 0.1 M phosphate buffer, dehydrated in ascending ethanol series, and embedded in Spurr’s resin. Sections (1 μm) were cut with a Leica EM UC6 ultramicrotome, then mounted and stained with methylene blue and toluidine blue. All light microscopy was conducted using a Leica DMI3000 inverted microscope.

## Results

### Identification of genes encoding JH biosynthetic enzymes and Vg in *D*. *punctata*

*CYP15A1* was previously identified in *D*. *punctata* and the genes encoding HMGS, MK, FPPS and FOLD were present in a CA EST database of *D*. *punctata* [19,20]. We confirmed the sequence of *HMGS*, *MK*, *FPPS* and *FOLD*. For the remaining enzymes in the JH biosynthetic pathway, we aligned (predicted) orthologous sequences from different insect orders and designed degenerate primers based on conserved domains. We succeeded in cloning an additional 5 (partial) sequences for enzymes in the conserved mevalonate pathway: *Thiol*, *HMGR*, *PMK*, *PPMD* and *IPPI* and one extra sequence encoding an enzyme in the JH-specific part of the pathway, *JHAMT*. The complete sequences for *HMGR* and *JHAMT* were obtained. The ORFs of *HMGR* and *JHAMT* encode proteins of 825 and 274 amino acids in length, respectively. Amino acid alignments of these proteins with known insect orthologs are given in S1 Fig.. Moreover, a partial sequence of *Vg* was cloned from *D*. *punctata* adult female fat body. The sequences for the genes were deposited in NCBI’s GenBank and received accession numbers: *Thiol* (KJ188021), *HMGS* (KJ188022), *HMGR* (KJ188023), *MK* (KJ188024), *PMK* (KJ188025), *PPMD* (KJ188026), *IPPI* (KJ188027), *FPPS* (KJ188028), *FOLD* (KJ188029), *JHAMT* (KJ188030), *CYP15A1* (AY509244) and *Vg* (KJ188031).

### Tissue specificity and developmental transcript profiles during the first gonadotropic cycle are consistent with roles of these genes in JH biosynthesis

**Fig 2:**
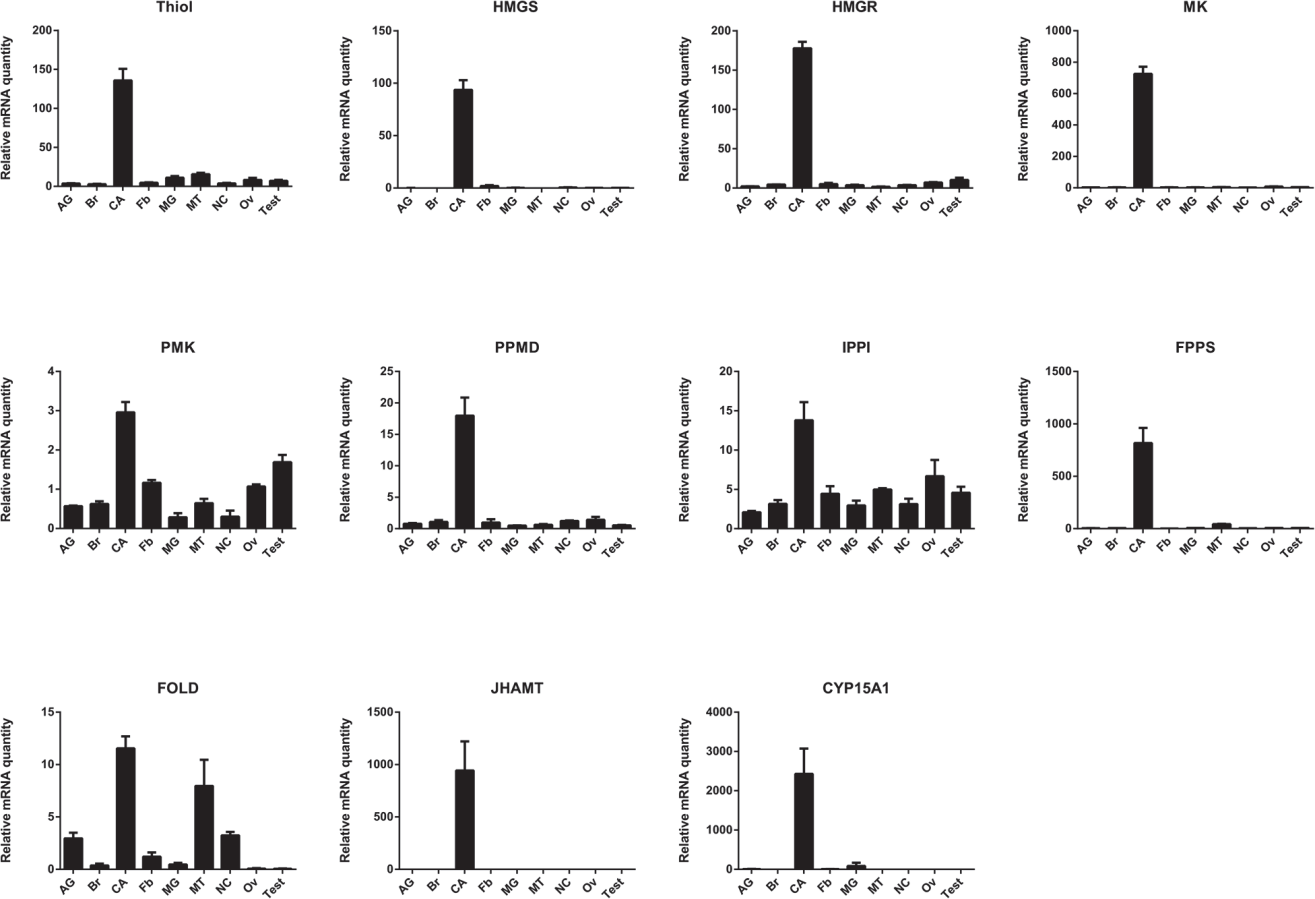
Tissue specific expression of genes encoding JH biosynthetic enzymes. All tissues were dissected from day 4 mated adult females, except accessory glands (AG) and testes (Test) were from day 4 adult male. Abbreviations on the X-axis: Brain (Br), corpora allata (CA), nerve cord (NC), midgut (Mg), Malpighian tubules (MT), fat body (Fb), and ovary (Ov). Bars represent the mean of three biologically independent pools of ten animals run in triplicate and normalized to *Tubulin* and *EF1α*. Vertical error bars indicate SEM.

Using q-RT-PCR, the relative transcript levels of 11 genes in the JH biosynthetic pathway were determined in several tissues of day 4 mated females and males. Relative transcript levels for the *Diploptera* orthologs of genes in the JH pathway were normalized to transcript levels of the reference genes *EF1a* and *Tubulin* [23]. To represent the tissue distribution data for the 11 enzyme-encoding genes in one figure, the transcript levels were normalized to the transcript level of *PMK* measured in the calibrator sample. The orthologous genes encoding enzymes in the JH biosynthetic pathway were most highly expressed in the CA, which is consistent with the functions of the enzymes (Fig. 2). Most genes appear to be exclusively expressed in the day 4 female CA: *Thiol*, *HMGS*, *HMGR*, *MK*, PPMD, *FPPS*, *JHAMT* and *CYP15A1*; whereas a few show a broader tissue distribution: *PMK*, *IPPI* and *FOLD*. These latter 3 appear to be expressed not only in nervous tissues but also in peripheral tissues such as the ovary, fat body and Malpighian tubules. Moreover, there are 1000-fold differences in relative mRNA levels of the enzyme-encoding genes, with *PMK*, *PPMD*, *IPPI* and *FOLD* being present in low abundance, *Thiol*, *HMGS* and *HMGR* being of intermediate abundance and *MK*, *FPPS*, *JHAMT* and *CYP15A1* being represented in high abundance (S2 Fig.).

**Fig 3:**
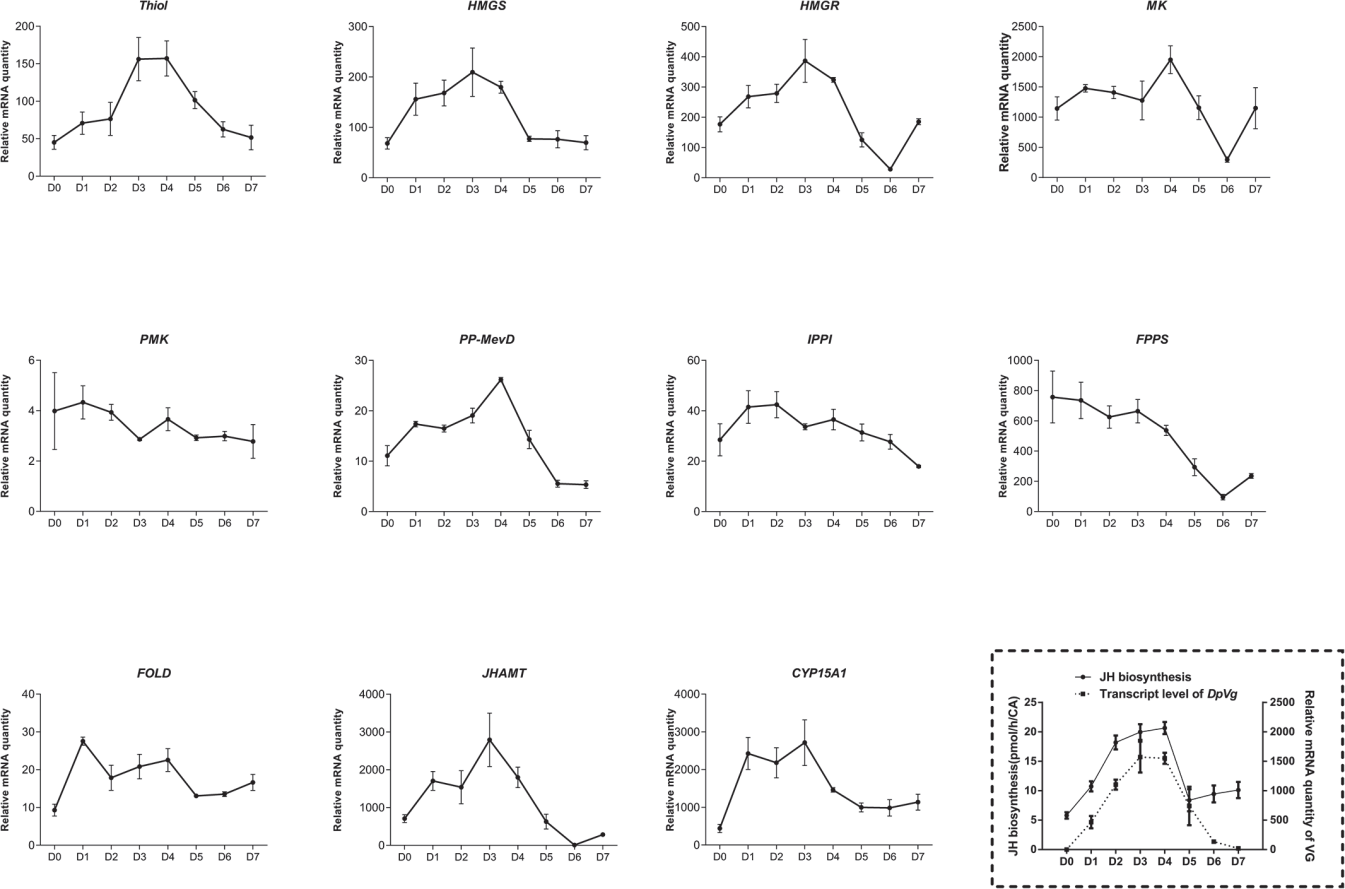
Developmental expression of genes encoding JH biosynthetic enzymes during the first gonadotrophic cycle of *D*. *punctata*. Measurements were taken every day during the cycle (day 0 to day 7 after the final molt). Bars represent the mean of three biologically independent pools of ten animals run in triplicate and normalized to *Armadillo* and *EF1α*. Vertical error bars indicate SEM. Inset at the right bottom shows JH biosynthesis per individual CA (n = 12) and the transcript level of *DpVg* in fat body during the first gonadotrophic cycle [27]. Vertical error bars indicate SEM.

Our next step was to follow the relative transcript levels in the CA of mated females throughout the first gonadotropic cycle. Target gene expression was normalized to transcript levels of *EF1a* and *Armadillo* according to a previous study [23]. In general, the expression of the 11 genes is correlated with the *in vitro* rates of JH biosynthesis in the CA and relative mRNA levels for *DippuVg* measured in the female fat body (Fig. 3). The relative mRNA levels in the CA for most of the genes of interest were low at the beginning of the adult female stage when JH biosynthesis is low, rose during the vitellogenic portion of the first gonadotropic cycle reaching a peak on day 3–4 and thereafter began to decline on day 5, attaining a low level during oviposition on day 7. However, there appear to be three exceptions to this pattern: *PMK*, *IPPI* and *FPPS*. Relative mRNA quantities for *PMK* and *IPPI* were very low compared to other genes and did not display dramatic changes during the first gonadotropic cycle. *FPPS* transcripts were high on day 0, remained high until day 4 and then declined on day 5 when vitellogenesis slows.

### Addition of JH precursors stimulates JH biosynthesis in CA with low JH biosynthetic activity in vitro

**Fig 4:**
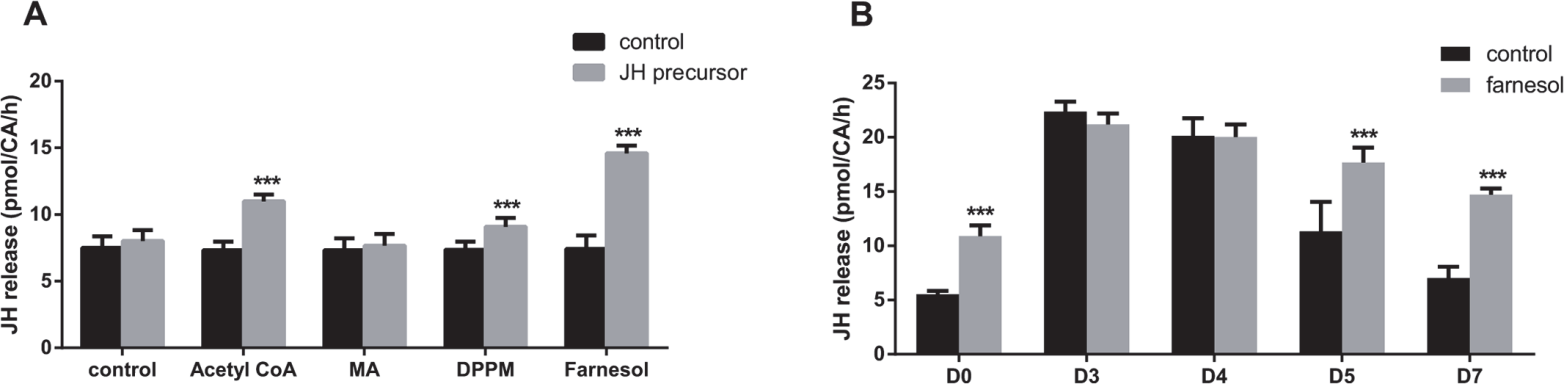
The effect of JH precursors on JH biosynthesis by CA from mated female *D*. *punctata*. JH biosynthesis was determined in CA that were first incubated in medium TC199 (control), and then in medium with JH precursor (treatment). (A) JH precursors stimulate JH biosynthesis by CA from day 7 mated female cockroach, *D*. *punctata*. 100μM of JH precursor was added to the medium during the second incubation. (B) The sensitivity of CA to JH precursors during the first gonadotrophic cycle. 40μM farnesol was added during the second incubation. Values represent mean ± SEM (n≥10). Significant differences are indicated ***p < 0.001.

Previous studies suggested that JH synthesis is controlled by the rate of flux of isoprenoids in *A*. *aegypti* [11]. To determine the role of other JH precursors in regulating JH biosynthesis, we tested the rate of JH biosynthesis with the addition of JH precursors in the early steps of mevalonate pathway or the addition of farnesol. The addition of acetyl CoA, DPPM or farnesol to the incubation medium had a significant stimulatory effect on JH biosynthesis, whereas MA did not (Fig. 4A). For the first time, we demonstrated that acetyl CoA, as the first precursor in the JH biosynthetic pathway, was able to stimulate JH biosynthesis. The rank order of the stimulatory effects of the different JH precursors on JH biosynthesis is as follows: farnesol > acetyl CoA > DPPM > MA.

We also evaluated the sensitivity of CA to farnesol during the first gonadotrophic cycle. CA were dissected from day 0, 3, 4, 5, and 7, and incubated with medium containing 40 μM farnesol. On days 3 and 4, when the CA show high JH biosynthetic activity, the addition of farnesol had no effect on *in vitro* JH biosynthesis (Fig. 4B). On the other hand, JH biosynthesis in CA of day 0, 5 and 7 mated females was significantly increased.

### Injection of *HMGR-JHAMT* dsRNA resulted in a significant downregulation of the target genes but also of other genes in the JH biosynthetic pathway

**Fig 5:**
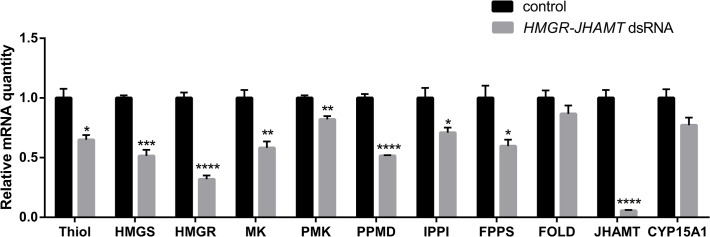
Efficiency of *HMGR*-*JHAMT* RNAi-mediated knockdown and the effect of silencing on the transcription of the other genes encoding enzymes in the JH biosynthetic pathway in day 4 mated female *D*. *punctata*. The data represent averages of 3 pools (7 pairs of CA per pool), run in triplicate using q-RT-PCR and normalised to *Tubulin* and *EF1a* transcript levels. Values represent mean ± SEM. Significant differences are indicated by asterisks (*p < 0.05, **P < 0.005, ***p < 0.001, ****p < 0.0001).

A systemic RNAi response was observed following injection of *HMGR*- and *JHAMT* dsRNA into animals every other day during the first gonadotropic cycle. Relative transcript levels were measured in the CA of day 4 animals using q-RT-PCR. A significant knockdown of 64% and 94% was measured for *HMGR* and *JHAMT*, respectively. Off-target effects were investigated by checking the Ct value of housekeeping genes (S2 Table). Moreover, two different dsRNA constructs were used to eliminate off-target effects (primers listed in Table 2). Both constructs yielded a similar phenotype.

q-RT-PCR was used to determine the relative mRNA levels of the other genes involved in the JH biosynthetic pathway using the control and treated CA described above. A significant reduction in relative expression levels was observed for all genes encoding the enzymes directly involved in the pathway, with the exception of the genes encoding FOLD and the epoxidase, CYP15A1 (Fig. 5).

### Silencing *HMGR* and *JHAMT* resulted in a significant reduction in rates of JH biosynthesis and slows ovarian development

**Fig 6:**
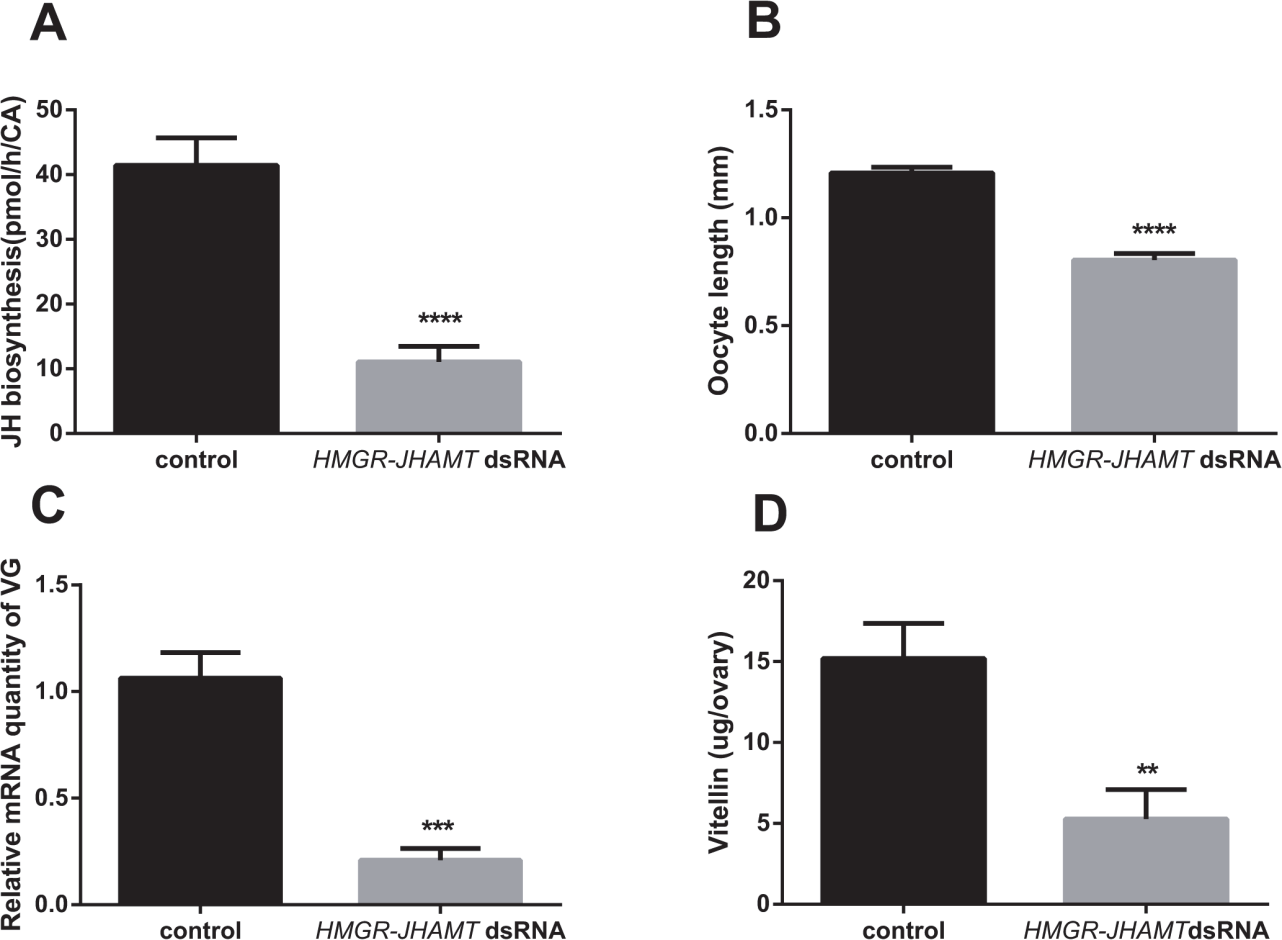
JH regulates ovarian development: (A) The application of *HMGR*-*JHAMT* dsRNA results in a dramatic decrease of JH biosynthesis (n = 16). (B) Oocyte length in *HMGR*, *JHAMT* dsRNA-treated animals compared to control animals (n≥27). (C) Relative *Vg* mRNA levels in the fat body of *HMGR-JHAMT* dsRNA-treated animals compared to control animals. The transcription data represent averages of 3 pools (5 animals per pool), run in triplicate using q-RT-PCR and normalised to *Tubulin* and *EF1a* transcript levels. (D) Vitellin content measured by ELISA in *HMGR*-*JHAMT* dsRNA-treated animals compared to controls (n = 9). Animals were dissected on day 4 after the final molt in control and treated groups. Values represent mean ± SEM. Significant differences are indicated by asterisks (**P < 0.005, ***p < 0.001, ****p < 0.0001).

The downregulation of *HMGR* and *JHAMT* resulted in a 73% reduction in the rates of JH biosynthesis in the CA of day 4 adult females as measured using the *in vitro* RCA (Fig. 6A). To confirm the role of JH in inducing *Vg* transcription in the fat body and uptake in the developing basal oocytes, fat body and ovaries were dissected from day 4 control and treated adult cockroaches. The basal oocyte length was significantly decreased following silencing of *HMGR* and *JHAMT*. The average oocyte length in the *HMGR*-*JHAMT* RNAi animals measured 0.8 mm compared to the control of 1.21 mm (Fig. 6B). The transcript level of the *Vg* in the fat body of the *HMGR*-*JHAMT* dsRNA-treated animals was reduced 80% (Fig. 6C). In addition, the knockdown of *HMGR*-*JHAMT* mRNA resulted in a significant reduction in vitellin content compared to the controls (Fig. 6D).

**Fig 7:**
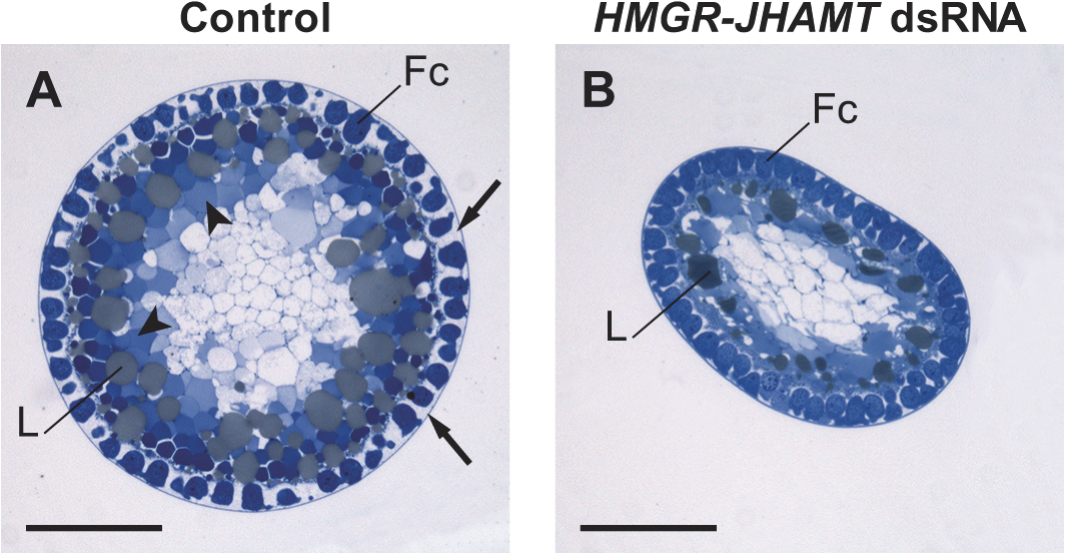
Silencing *HMGR*-*JHAMT* affects patency. Transverse sections of the basal oocytes from day 4 control and *HMGR*-*JHAMT* dsRNA-treated animals. Follicle cells (Fc), intercellular gaps (arrows), yolk spheres (arrowheads) and lipid spheres (L) are indicated. Scale bars represent 200 μm.

We also studied the histology of the developing basal oocytes in control and treated females. Control oocytes were fully patent on day 4, showing large intercellular spaces between the follicle cells and the clear presence of yolk spheres. In the *HMGR*-*JHAMT* RNAi animals, on the other hand, patency was not fully induced and as a result, yolk was not deposited in these oocytes (Fig. 7).

## Discussion

### Sequence of genes encoding enzymes in the JH biosynthetic pathway

Through *in silico* data mining and degenerate primer PCR and RACE, we have successfully characterized 11 of the 13 enzyme-encoding genes in the JH biosynthetic pathway of *D*. *punctata*. Cheng *et al* [9] showed that enzymes in the early steps of the mevalonate pathway were generated by single copy genes in many insects, whereas genes encoding FPPS and enzymes in the JH-specific pathway probably underwent gene duplication in Lepidoptera. In *D*. *punctata*, only a single copy of these genes was identified. However, at this point, the existence of multiple copies of several of these genes cannot be ruled out and this issue will have to await the assembly of the *Diploptera* genome.

The full-length *HMGR* and *JHAMT* were cloned from *D*. *punctata* CA. *HMGR* encodes a protein of 825 amino acids in length. Following a conserved domain search, HMGR was observed to contain the typical conserved motifs found in members of the HMG-CoA reductase superfamily of proteins. *JHAMT* encodes a protein of 274 amino acids in length and contains the motifs typically found in AdoMet-dependent methyltransferases. Still missing are sequences for farnesyl diphosphate pyrophosphatase (FPPP) and farnesal dehydrogenase (FALD). Molecular analysis of these genes was long hampered as a consequence of the minute size of the CA but using a newly developed sensitive assay for measuring JH precursor pools employing fluorescent tags, the genes encoding FPPP and FALD were recently characterized in *A*. *aegypti* [14,15,29]. These were, however, not included in our study because the availability of only dipteran sequences made their characterization in the phylogenetic basal *D*. *punctata* problematic and therefore remain uncharacterized in this species to date.

### Transcription of enzymes in the JH biosynthetic pathway partly regulates JH biosynthesis

The 11 orthologs of JH biosynthesis genes cloned in our model *D*. *punctata* are predominately expressed in the CA, and their relative transcript levels correlate well with the rates of JH biosynthesis (Figs. 2 and 3). Our results suggest that CA are highly specialized tissues for the biosynthesis of JH, and genes identified in our study are encoding functional enzymes in the JH pathway. Enzymes in the mevalonate pathway are responsible for not only JH production but also for the production of other terpenoids such as defensive secretions and pheromones (as reviewed by [4,30]). It is therefore of interest that all mevalonate enzyme-encoding genes except *PMK* and *IPPI* are exclusively expressed in the CA with trace amounts in other tissues. This can be explained by the strikingly high rates of JH biosynthesis in the CA of day 4 adult female *D*. *punctata*. For the later enzymes involved in the JH specific steps of the pathway, the transcription of *JHAMT* and *CYP15A1* is CA-specific, whereas *FOLD* is expressed in many tissues. Similar results were observed in *A*. *aegypti* and *A*. *mellifera* [11,12,31]. Because of the multiple functions of farnesol, the oxidation of farnesol to farnesal does not appear to be a JH biosynthesis-specific reaction [31].

Changes in rates of JH biosynthesis during the first gonadotropic cycle of *D*. *punctata* are dynamic, corresponding to specific reproductive events (see below) and are therefore tightly regulated. The transcript levels of enzyme-encoding genes in JH biosynthesis are highly coordinated with the rates of JH biosynthesis (Fig. 3), suggesting that at least part of the regulation of JH biosynthesis involves coordinated changes in the transcription of the genes in the biosynthetic pathway, in agreement with studies performed in *A*. *aegypti* [11] and *B*. *mori* [8], although the ability of each gene to regulate JH biosynthesis appears to differ. In addition, the expression patterns of *HMGS* and *HMGR* mRNA were similar to the patterns of their enzyme activities [32,33]. Although the transcription of most genes in the biosynthetic pathway showed a correlation with the JH biosynthesis, there are a few exceptions, including PMK, IPPI and FPPS. PMK and IPPI, which are expressed in multiple tissues (Fig. 2), and may not be specifically regulated in the CA. For FPPS, 3 FPPS homologs were found in *B*. *mori* [10], and 7 in *A*. *mellifera* [12]. Although we currently have identified only 1 FPPS gene in *D*. *punctata*, additional FPPS genes may exist in the CA that regulate JH biosynthesis.

### Effect of exogenous JH precursors on rates of JH biosynthesis

We have found that JH precursors are able to stimulate JH biosynthesis (Fig. 4A), which suggests that the rate of JH biosynthesis is not only controlled by transcription of the genes in the pathway, but also by the flux of substrates in the pathway. Farnesol stimulates JH biosynthesis by CA from day 0, 5 and 7 mated females. However, application of farnesol to CA showing high JH biosynthetic activity (day 3 and 4) does not result in a significant change in JH biosynthesis (see Fig. 4B). These results suggest that during the first gonadotrophic cycle of *D*. *punctata*, the supply of this precursor is rate-limiting in CA with showing low JH biosynthetic activity, whereas there are other factors, including nutrients and neurotransmitters that control JH production at high rates of JH biosynthesis. Nevertheless, 14 JH precursors are involved in the JH biosynthetic pathway, and each JH precursor connects to the ‘upstream’ and ‘downstream’ enzymes. Many factors could affect the stimulatory effect of JH precursor in JH production, including cell permeability of the added compounds, the activity of upstream/downstream enzymes, and the size of other JH precursor pools.

### A feedback mechanism involved in the regulation of the transcription of JH biosynthetic genes

HMGR and JHAMT have been studied in the JH pathway since they are representative enzymes involved in the well-conserved mevalonate pathway and in the JH-specific portion of the pathway respectively [6,34–39]. We therefore chose to silence *HMGR* and *JHAMT* on a posttranscriptional level using RNAi during the first gonadotropic cycle of *D*. *punctata*. RNAi trials have shown that fewer injections of *HMGR* or *JHAMT* dsRNA and with lower concentrations of construct, did not result in a significant silencing of our target gene mRNA levels (data not shown). We therefore conclude that the RNAi response in *D*. *punctata* appears to be systemic, but is not as effective as in another hemimetabolous insect model, the desert locust, *Schistocerca gregaria* in which smaller quantities of the *JHAMT* dsRNA construct induced a longer downregulation of the same target gene during the first gonadotropic cycle [6]. A further in-depth study on the RNAi machinery *D*. *punctata* should be performed to explain the differences in sensitivity compared to other hemimetabolous insect species.

JH biosynthesis is controlled by many factors including neuropeptides (allatostatins, allatotropins, short neuropeptide F) [40,41], neurotransmitters (octopamine, dopamine and glutamate) [42–44] and JH itself [1,16]. Topical application of JH or a JH analog resulted in a suppression of JH biosynthesis, which suggests a negative feedback regulation of CA in *D*. *punctata* [45]. In higher animals, cholesterol, the bulk end-product of mevalonate pathway, shows a feedback regulation on the transcription and post-transcription of enzymes in the mevalonate pathway [30,46,47]. Later studies showed that farnesol, an intermediate product in the JH biosynthetic pathway, is able to accelerate the degradation of HMGR, and the increase of mevalonate flux raises the activity of FPPP expressed in CHO cells [48,49]. In our study, regulating transcription of the JH biosynthetic enzymes seems to affect the entire biosynthetic pathway, rather than individual steps. The silencing of *HMGR* and *JHAMT* resulted in a significant decrease in the transcript level of several other genes in the pathway (Fig. 5). A possible explanation is that the accumulation of JH precursors as a result of RNAi treatment resulted in a feedback on the expression of other genes in the JH biosynthetic pathway to balance the size of JH precursor pools and the enzyme activity.

### The essential role of JH in reproduction

Our results clearly show that select silencing of genes encoding enzymes in the JH biosynthetic pathway effectively reduces JH biosynthesis *in vitro* (Fig. 6). As a well-known model for the study of the regulation of JH biosynthesis, *D*. *punctata* displays a consistent characteristic profile of JH production during the first gonadotropic cyclethat is closely correlated with specific reproductive events such as vitellogenesis and chorionation. Once JH production rises on day 2, vitellogenin synthesis in the fat body commences, and vitellogenin is taken up from the hemolymph and is incorporated into the developing basal oocytes. The elevated JH titer results in patency in the follicular epithelium of the basal oocytes, permitting the uptake of Vg and subsequent yolk formation [27,50]. On day 5, the spaces in the follicular epithelium close as rates of JH biosynthesis decline and *Vg* transcription in the fat body and circulating Vg levels decrease. At this point, choriogenesis begins and JH biosynthesis remains low until oviposition on day 7 [as reviewed by 16]. Our RNAi study has focused on day 4 of the first gonadotropic cycle, a time when JH titre is high in controls and animals are vitellogenic [51] as characterized by high *DippuVg* mRNA transcript levels in the fat body and JH-dependent induction of patency in the maturing basal oocytes. Following the reduction in JH production by the silencing of *DippuHMGR* and *DippuJHAMT*, mRNA levels of *DippuVg* in the fat body are significantly reduced, incorporation of vitellin into the oocytes is impaired and patency does not occur (Figs. 6 and 7). Previous reports have shown that in *S*. *gregaria*, oocyte length is significantly affected following treatment of females with *JHAMT* dsRNA [6]. RNAi was also used in the cotton bollworm *Helicoverpa armigera* to effectively silence *HMGR*, resulting in reduced Vg expression [38]. Each of these studies highlights specific aspects of JH-regulated reproductive events. The results described in the current paper confirm the central role played by JH during the female reproductive cycle of *D*. *punctata*.

JH biosynthesis is a fundamental process in regulating insect development, metamorphosis and reproduction [1]. Although the genes directly involved in JH biosynthesis have been characterized using molecular techniques in several insect species, there was no similar study in the hemimetabolous insects. Thus, our study is the first to characterize the majority of the genes directly involved in JH biosynthesis in a hemimetabolous insect and a well-known model for studying the physiology of JH. This paper now provides the molecular tools to study the regulatory mechanisms of JH production in *D*. *punctata* (for a first study, see [52]). In addition, the role of JH biosynthetic enzymes and the JH precursor supplies in regulating JH biosynthesis have been demonstrated, as has the critical function of JH as the master regulator of cockroach female reproduction.

## Supporting Information

S1 Fig Amino acid sequence alignment of *Diploptera* proteins with several functionally characterized orthologous insect proteins. (A) DippuHMGR with HMGR from the German cockroach *Blattella germanica* (GenBank accession number: CAA49628.1 [36], the honeybee *Apis mellifera* (GenBank accession number: XP_623118.1), the red flour beetle *Tribolium castaneum* (GenBank accession number: XP_973850.1), the mosquito *Aedes aegypti* (GenBank accession number: XP_001659923.1), the fly *Drosophila melanogaster* (GenBank accession number: NP_732900.1) and the silkworm *Bombyx mori* (GenBank accession number: BAF62108.1 [8]) (B) DippuJHAMT with JHAMT from the desert locust, *S*. *gregaria* (GenBank accession number: ADV17350.1 [6]), the red flour beetle *T*. *castaneum* (GenBank accession number: NP_001120783.1 [53]), the fly *D*. *melanogaster* (GenBank accession number: AB113579.1 [54]), the mosquito *A*. *aegypti* (GenBank accession number: ABD65474.1[39]), the silkworm *B*. *mori* (GenBank accession number: NP_001036901.1 [7]) and the honeybee *A*. *mellifera* (GenBank accession number: AGG79412.1).
(PDF)

S2 Fig Comparison of transcript levels of genes encoding JH biosynthetic enzymes in CA of day 4 mated female. Bars represent the mean of three biologically independent pools of ten animals run in triplicate and normalized to *Tubulin* and *EF1α*. Vertical error bars indicate SEM. Enzyme abbreviations are as in Fig. 1.
(EPS)

S1 Table (Degenerate) primer sequences for cloning of (partial) sequences for orthologous genes encoding JH biosynthesis enzymes in *D*. *punctata*. Gene name abbreviations are shown in Fig. 1.
(DOCX)

S2 Table The Ct value of housekeeping genes (EF1α and Tubulin) in the CA and fat body sample. (DOCX)
