# Supplementary material for: Characterization of the Juvenile Hormone Pathway in the Viviparous Cockroach, Diploptera punctata
Source: PLoS One. 2015 Feb 23;10(2):e0117291. doi: 10.1371/journal.pone.0117291 (PMC4338245; doi:10.1371/journal.pone.0117291)
Supplement: S1 Fig — (A) DippuHMGR with HMGR from the German cockroach Blattella germanica (GenBank accession number: CAA49628.1 [36], the honeybee Apis mellifera (GenBank accession number: XP_623118.1), the red flour beetle Tribolium castaneum (GenBank accession number: XP_973850.1), the mosquito Aedes aegypti (GenBank accession number: XP_001659923.1), the fly Drosophila melanogaster (GenBank accession number: NP_732900.1) and the silkworm Bombyx mori (GenBank accession number: BAF62108.1 [8]) (B) DippuJHAMT with JHAMT from the desert locust, S. gregaria (GenBank accession number: ADV17350.1 [6]), the red flour beetle T. castaneum (GenBank accession number: NP_001120783.1 [53]), the fly D. melanogaster (GenBank accession number: AB113579.1 [54]), the mosquito A. aegypti (GenBank accession number: ABD65474.1[39]), the silkworm B. mori (GenBank accession number: NP_001036901.1 [7]) and the honeybee A. mellifera (GenBank accession number: AGG79412.1). (PDF) [file pone.0117291.s001.pdf]

A.

```
BlageHMGR    1 M-VGRLFRAHGQFCASHPWEVIVATLTLTVCMLTVDQ-------------RP-LGLPPGWGHNC----------------------------------ITLEEYNAADMIVMTLIRCVAVLYSYYQFCHLQKLGSKYILGI
DippuHMGR    1 M-VGWLFLAHGQFCASHPWEVIVATFTLTACMLTVDP-------------HP-LGLPPGWGRKNC----------------------------------ISLEEYNAADVIVMTLIRCVAVLYSYYQFCHLQKLGSKYILGI
ApimeHMGR    1 M-LTRLFEIHGRFCAGHPLEVIVTTFTLTACILNMETG----------NGHPSVPLTAHCGPGR----------------------------------CNSDDLNAADVIVMTIIRCLAILFTYHQFRNLQKLGSKYILGI
TricaHMGR    1 M-TTRLFRAHGEFCATHPWEVIVATLTLAACMLTVDQQ----------HPAPPPKPTLRYCAEC----------------------------------LQEAEYNAADLIVMTLIRCLAVLYCYYQFCNLEKLGSKYILGI
AedaeHMGR    1 MKVGRLFRAHGEFCASHPWEVIVALVTLTACIITFDKG----------SADPFQQSSSRSGSGRPC----------------PSWRFSSVSPDEAFRCEEVEQNGIDVILMTIVRCSAILYCYYHFCNLQKLGSKYILGI
DromeHMGR    1 M-IGRLFRAHGEFCASHPWEVIVALLTITACMLTVDKNNTLDASSGLGTATASAAAAGGSGSGAGSGASGTIPPSSMGGSATSSRHRPCHGWSQSCDGLEAEYNAADVILMTIVRCTAVLYCYYQFCSLHRLGSKYVLGI
BommoHMGR    1 M---KVWGAHGEFCARHQWEVIVATLALLACAASVERHG----------PGNRSEHCAGWARACP----------------------------------GLEAEYQAADAVIMTFVRCAALLYAYYQVLNLHKIASKYLLII

BlageHMGR   93 AGLFTVFSSFVFSSSVINFLGSDVSDLKDALFFFLLLIDLSKATVLAQFALSSRSQDEVKHNIARGIAMLGPTITLDTVVETLVIGVGMLSGVRRLEVLCCFACMSVIVNYVVFMTFYPACLSLILELSRSGESGRPAWH
DippuHMGR   93 AGLFTVFSSFVFSSSVINFLGSDISDLKDALFFFLLLIDLSKATLLAQFALSSCSQDEVKHNIARGIAMLGPTITLDTVVETLVIGVGTLSGVRRLEVLCCFACMSVIVNYIVFMTFYPAFLSLILELARSGEGGRPAWH
ApimeHMGR   97 AGLFTVFSSVVFTSSVVNFGRSDISDLKDALFFFLLIIDLSKAAVLAQLALSSRNKEEVRANIARGMSLLGPTITLDTLVETLLISIGSLSGVRRLEILCSFACLGVVVNYIVFMTFYPACLSLILELSRETNTMKPLSA
TricaHMGR   97 AGLFTVFSSFVFTSTVLNLLWIDVSDLKDALFFFLLLIDLSKAAMLAQSALSASNQEEVKSNIARGMAVLGPTITLDTIVETLVIGVGTLSGVHRLEMLSYFACLSVLVNYIVFMTFYPACLSLILELSRTN---IYGN
AedaeHMGR  115 AGLFTVFSSFIFTSTVINFLGSEVSDLKDALFFFLLLIDLSKAAILAQLALCGSSQSEVTMNIARGMEILGPAISLDTLVETLVIGVGTLSGVQRLEILSGFAVLSAIVNYIVFMTFYPACLSLILDLSRNAGNLIQKNK
DromeHMGR  140 AGLFTVFSSFIFTTAIIKFLGSDISELKDALFFLLLVIDLSNSGRLAQLALSGSNQAEVTQNIARGLELLGPAISLDTIVEVLLVGVGTLSGVQRLEVLCMFAVLSVLVNYVVFMTFYPACLSLIFDLSRSGVDMSVVRE
BommoHMGR   96 AGVFSTFASFIFTSALASLFWSELASIKDAPFLFLLVADVARGARMAKAGWSAG--EDQGKGVGRALSLLGPTATLDTLLAILLVGVGALSGVPRLEHMCTFACLALLVDYIVFITFYPACLSLVADFATNRK---EIAH

BlageHMGR  233 DKS--LIIKALHEEDQKPNPVVQRVKVIMSAGLMLVHAHRWVRCLSIALWPDLTSLRYFCTHCDTGVSYSRWSFASEGEELPTVKLVTG--DSVVNSNST----DDAQLHYYIMRWLTVSADHIVILILLLALAVKFVFF
DippuHMGR  233 DRS--LIMKALHEEDQKPNPVVQRVKVIMSAGLMLVHAH----------------------------------SRWSFASEGEELPIVNLVTG--DSVVITNST----QDAQIHDYIMRWLTVSADHIVILILLLALAVKFVFF
ApimeHMGR  237 DKI--FMMHPLDEEDQKPNPVVERVKLIMIAGLFVVHAN----------------------------------SRFKSE-ESEDTVEGKVSST--NSHVIVNSYNETEDSSEVKGYLMNWLSVSADNIVILILLLALAIKFIFF
TricaHMGR  234 KQS--LIARALKEEDHKSNPVVQRVKLIMSAGLMLVHAR----------------------------------SRWPFK--EDDVENIRPLVV--EQHMTLNRT----EDTTLHEYIMKWVTVSADHIVILILLLALVVKFVFF
AedaeHMGR  255 KEN--LLARVLTEEDQKPNPVVQRVKLIMSSGLMIVHVL----------------------------------SRLAISEKDSDTAEHIIASHSHEHLAAMNKT----EPNEISEFIMRWLSISTEQIVTYILIIIALGVKFVFF
DromeHMGR  280 KAKGSLLLKSLTEEEQKANPVLQRVKLIMTTGLMAVHIY----------------------------------SRVAFSGSDYDAVDKTLTPT-LSLNVSNNRT----ESGEIADIIIKWLTMSADHIVISIVLIAIVVKFICF
BommoHMGR  231 DSP-------FSEEDLKPNPVVQRVKMIMAAGLLCVHLT----------------------------------SRWPWS------ANHGIIEG--PIDASIPVP----HDNILLHSYVKWFSVSADYIVIATLLCALIIKFIFF

BlageHMGR  365 ETRDELTTTRGMDGWVEVSSP------VEHKYVQTE-----------------------------------QPSCSAP------EQPLEEPPASN------------RSIDECLSVCK-SDVGAQ--ALSDCEVMALVTS-
DippuHMGR  335 EVKDELNTTRGMDGWVEVSSP------VEHKFVQTD-----------------------------------QVCWELPSSS---QEPDEQPLPCD------------RPVQECLAICK-SDAGAN--SLSDSEVMSLVSS-
ApimeHMGR  342 EDKEDIAKQLQFKVEDDTEEK------IENEYMKEKKFEIEYVKDKENENEDMNFSFLTTKMPFKLSFAKMQTISIPWIDGKEEQIDEKQCTVNTSNDKNLFSQIPRSVEECLKIYK-SELGAN--GLTDEEVIQLVKN-
TricaHMGR  334 ENKEELAEQLRAHISTESVDPGKKDNRFKMPLIKTQSFFLTNNTKED---------------------SACEDKEVQTDIGRLESEFEKLPAGN-----DVSVKTCRSLEECLKIYNDSNLGGA--ALSDDEVILLVKN-
AedaeHMGR  359 D-RHNLSDQILLSVANEAAAAAAAAAAAAQKQKQLELELERPQPVATFT--------LAETVPVEEKATQSELCLLPGRGQRAKSMGADDELNLDDLEDELVEREPRPLEMCLKILNETDDGAV--GLTDEEIKMIVRAG
DromeHMGR  385 DNRDPLPDQLRQSGPVAIAAKASQTTPIDEEHVEQEKDTENS---------------------------AAVRTLLFTIEDQSSANASTQTDLLPLRHRLVGPIKPPRPVQECLDILNSTEEGSGPAALSDEEIVSIVHAG
BommoHMGR  322 EEQRNWVYDMDDMTVKEVINDTDLS--RKPKFSVGD-----------------------------------DSNSEVSTQTDEAGNVEDMEWPTLSPSSSASKLNAKKRPMVECLELYR---SGACT-SLSDEEVIMLVEQ-

BlageHMGR  443 -GHIAGYQLEKVVRNPERGVGIRRQILTKTADL-KDALDNLPYKNYDYLKVMGACCENVIGYMPVPVGVAGPLNLDGRLVHVPLATTEGCLVASTNRGMRALMRCGVTSRIVADGMTRGPVVRFPNIDRASEAMLWMQVP
DippuHMGR  416 -GHVAAYQLEKMVGDPERGVGIRRKILTQKADL-KDALDNLPYKNYDYTKVMGACCENVIGYMPVPVGVAGPLKLDGCLVYVPLATTEGCLVASTNRGSRALMRCGVTSRIVADGMTRGPVVRFPNIERASEAMLWMQAA
ApimeHMGR  472 -NHIAAYQLEKAVGDMERGVEIRRFIIGEAGNF-LDYLSNLPYKDYDYSKVLGACCENVMGYVPVPLGIAGPLLLDGELYYVPMATTEGCLVASTNRGSRALLKCGVTSRVVADGMTRGPVVRFPNIVRASEAMAWMQDP
TricaHMGR  445 -KHIPAYQIEKAVDDPERGVGIRRKILAREGNF-SEALTDLPFRNYDYAKVMGACCENVIGYMPVPVGYAGPLNLDGRHVYVPMATTEGCLVASTNRGCRALLDCGVTSRVVSDGMTRGPVVRFPSITKASEAMSWMKCS
AedaeHMGR  488 NGYCPLYKIETVIGDAERGVKIRRDMIQKEANLPANAFKHLSYKNYDYSKVMNACCENVLGYVQIPVGYAGPLLLDGVRYYVPMATTEGALVASTNRGCKAISTRGVTSFVEDIGMTRAPCIKFPNVLRAAQAKRWMETP
DromeHMGR  499 GTHCPLHKIESVLDDPERGVRIRRQIIGSRAKMPVGRLDVLPYEHFDYRKVLNACCENVLGYVPIPVGYAGPLLLDGETYYVPMATTEGALVASTNRGCKALSVRGVRSVVEDVGMTRAPCVRFPSVARAAEAKSWIEND
BommoHMGR  422 -SHIPMHRLEAVLEDPLRGVRLRRRVIASRFNN-ETAIKQLPYLNYDYSKVLNACCENVIGYIGVPVGYAGPLVVDGKPYMIPMATTEGALVASTNRGAKAIGSRGVTSVVEDVGMTRAPAVKLPNVVRAHECRQWIDNK

BlageHMGR  581 YNFEQIKKNFDSTSRFARLSKIHIRVAGRHLFIRFIATTGDAMGMNMLSKGTEVALAYVQQVYPDMEILSLSGNFCTDKKPAAVNWIEGRGKSVVCEAIVPADIIKSVLKTSVQALMDVNITKNLIGSAVAGSIGGFNAH
DippuHMGR  554 QNFEAMKKHFDSTSRYARLSKIHIRVAGRHLFIRFVATTGDAMGMNMLSKGTEVALSFVQQMFPDMEILSLSGNFCTDKKPAAVNWIEGRGKSVVCEAVVPADVVSSVLKTSVQALVDLNITKNFYGSAIAGSVGGNNAH
ApimeHMGR  610 DNFKEMKNSFNLTSRFARLTKINIRIAGRHLFIRFIATTGDAMGMNMLSKGTEKSLNTVKEHFPDMEILSLSGNFCTDKKPAAVNWVCGRGKSVVCEAVVPADIVTNVLKTSVHALVDVNISKNMIGSAIAGSIGGFNAH
TricaHMGR  583 QNFEAMKQQFDSSSRFARLSKLLIKIAGRHLFVRFEAKTGDAMGMNMVSKGTEMSLKYVQKQFPEMEILSLSGNFCTDKKPAAVNWIEGRGKSVVCEAIIPSEIVKKVLKTSTPALVDVNNSKNMIGSAVAGSIGGFNAH
AedaeHMGR  628 ENFAVIKKAFDSTSRFARLQEIHIAMDGPILYARFRALTGDAMGMNMVSKGSEMALREVHRSFPDMQIISLSGNFCSDKKPAAINWIKGRGKRVICEAIVPADKLRTILKTNARTLVQCNKLKNMTGSAVAGSIGGNNAH
DromeHMGR  639 ENYRVVKTEFDSTSRFGRLKDCHIAMDGPQLYIRFVAITGDAMGMNMVSKGAEMALRRIQLQFPDMQIISLSGNFCCDKKPAAINWIKGRGKRVVTECTISAATLRSVLKTDAKTLVECNKLKNMGGSAMAGSIGGNNAH
BommoHMGR  560 ENYALLKEAFDSTSRFARLQEIHVGVDGATLYLRFRATTGDAMGMNMVSKGAENALKLLKTFFRDMEVISLSGNYCSDKKAAAINWIKGRGKRVVCETVISSENLRTIFKTDAKTLSRCNKIKNLSGSALAGSIGGNNAH

BlageHMGR  721 AANIVTAIFIATGQDPAQNVGSSNCMTLMEPWGEDGKDLYVSCTMPSIEIGTIGGGTVLPPQAACLDMLGVRGANEMCPGENANTLARIVCGTVLAGELSLMSALAAGHLVKSHMRHNRSSVSTS----------GSEPS
DippuHMGR  694 AANIVTAIFIATGQDPAQNVGSSNCITIMEPWGDDKKDLYVSCSMPSIEIGTVGGGTILPPQAACLDMLGVKGANAVCPGENANMLARIVCGTVLAGELSLMSALAAGHLVKSHLRHNRSSVTTS----------GSEPS
ApimeHMGR  750 AANIVTAIFIATGQDPAQNVGSSNCMTLMEPWGTDGSDLYVSCTMPSIEIGTVGGGTILPAQGACLSILGVKGAHSDEPGENASRLARIVCATVLAGELSLMAALTAGHLVKSHLRHNRSSTTVTNAMSVPQKYTGTKLS
TricaHMGR  723 AANIVTAIYIATGQDPAQNIGSSNCMTLMEPWGETGEDLYVSCTMPSIEIGTVGGGTVLPAQSSCLEMLRVKGSHPDCPGENASQLARIVCGTVLAGELSLMAALTAGHLVRSHLRHNRSSTTIP-----------EEFS
AedaeHMGR  768 AANMVTAIYIATGQDPAQNVTSSNCSTNMEPYGDNGEDLYMTCTMPSLEVGTVGGGTGLPGQGACLEMLGVRGAHPTHPAENSKQLARVICATVMAGELSLMAALVNSDLVKSHMRHNRSSVAVNPG----LPATAQLQS
DromeHMGR  779 AANMVTAVFLATGQDPAQNVTSSNCSTAMECWAENSEDLYMTCTMPSLEVGTVGGGTGLPGQGACLEMLGVRGAHATRPGDNAKKLAQVICATVMAGELSLMAALVNSDLVKSHMRHNRSSIAVN-------------S
BommoHMGR  700 AANMVTAIFIATGQDPAQNVTSSNCSTNMEAYGENGEDLYVTCTMPSLEVGTVGGGTVLTGQGACLEILGVKGAG-TRPAENSARLASLICATVLAGELSLMAALVNSDLVKSHMRHNRSTLNVQ------------TAN
```

```
BlageHMGR  851 TP--------ACKS-------------
DippuHMGR  824 KS-------------------------
ApimeHMGR  890 VPNLLQPVQNVCKGLLEKS--------
TricaHMGR  852 QNRYHIP---PCKDI------------
AedaeHMGR  904 APSLLTACNSSSSGSSSSIGTSLSAKQ
DromeHMGR  905 ANNPLNVTVSSCSTIS-----------
BommoHMGR  827 VEPYTVALKVPPS--------------
```

B.

```
SchgrJHAMT    1 MDKAELYSRSNGLQRWEASAALEAAWPALRWPAPP-LRVLDVGCGAGDVTVDLLLPRLPP-HTQLVGTDVSAAMVEHAAELYGAAHPGLSFQLLDIADPDIDASPVYQLAPFDKIFSFFCLHWVPEQRQAAENLHRLLKP
DippuJHAMT    1 MHKAELYSSSHGLQKRDAAHALTEYLDHMTWR-PG-DRVLDVGCGPGFVTAQELMPRLPQDFAILVGTDVSHAMVQHATSTY--VQPKLKFAHLDISSTHIDK-ELWEPG-FDKIFSFYCLHWIPDQRTAVNNIYHLLRP
TricaJHAMT    1 MNKASLYSKYSGLQKNDASFVIDNYLRLIKWK-PN-ANILDIGSGDGNVIFELLLPKIPKHFAKFVGTDISEEMVLFAKNQCN--DPKIDFLQMDIS----ATIPPEFHEYFDHIFSFYCLHWVVEQRQAMKNIFDMLKP
DromeJHAMT    1 MNQASLYQHANQVQRHDAKLILDEFASTMQWRSDGEDALLDVGSGSGNVLMDFVKPLLP-IRGQLVGTDISSQMVHYASKHYQR-EERTRFQVLDIGCER-LPEELS--GRFDHVTSFYCLHWVQNLKGALGNIYNLLKP
AedaeJHAMT    1 MNKPNLYHRANGVQRRDAKEILDEHGHLLRWKEENEDSLLDIGCGSGDVLIDFVIPMVPPKRARVLGTDVSEQMVRFARKVHSD-VENLFFETLDIEGD--ISSFLNKWGCFDHITSFYCLHWVRSQRSAFSNIYNLMAP
BommoJHAMT    1 MNNADLYRKSNSLQKRDALRCLEEHANKIKWKKIG-DRVIDLGCADG-SVTDILKVYMPKNYGRLVGCDISEEMVKYANKHHG--FGRTSFRVLDIEGD--LTADLK--QGFDHVFSFYTLHWIRDQERAFRNIFNLLGD
ApimeJHAMT    1 MFLVEEYVKASTIQYRDAADIIGEFAEEMSEMKGK---CLDIGCGPGIVTKELILPNLSP-EAKLVGMDISRPMIEYAKNMYHD-EERLSFQLLDIET---MDLPKDTFDQFNNVLSFYCLHWCQNFRKAFDNIYKLLRP

SchgrJHAMT  139 GG-EVVLSLLAHCPIFSVYEGLAHKPQWKEYMEDARRFISPYHHSEDPAREMNELLCRAGFRVTLCTRQQRSFTFPGHSALIEAVTAVNPFVERLPETLQQEFLEDCMKEVLRQKLVSIEDDADSNNNSNGSNRSGNNAV
DippuJHAMT  135 AG-EALVLLMAKCPVFNVYTAQSNKPKWQQYMKDASRYISPYHQLKDPKSEFINIVEDVGFHVVDCDCRQNKFNYGTLERLKDAIKAVNPFMDRIPEELQEEYLNDCLSEARRIKCT------ESNNN-----------V
TricaJHAMT  133 GG-EMLLTFLASNPIYDIYERMAKSNKWGPYMNNLKKYISPYHHSEDPETELENLLKKEGFITHLCRVENRSYTFPSFSVLSKSVSAVNPFIKKLPENEIDTYIEDYLKEVRKLKTIT----IETCNN------------
DromeJHAMT  136 EGGDCLLAFLASNPVYEVYKILKTNDKWSTFMQDVENFISPLHYSLSPGEEFSQLLNDVGFVQHNVEIRNEVFVYEGVRTLKDNVKAICPFLERMPADLHEQFLDDFIDIVISMNLQQ-------GEN------------
AedaeJHAMT  138 NG-DCLLGFLARNPIFDIYDQLSNSAKWSMYMTDVDKYISPYQYCENPVGEIEEILSSVGFTKYKIHIADKIYVYEGIDSLKKAVQAVNPFSERMPLDLQEDFLNDYIAVVRRMSLSEN-----CCGN------------
BommoJHAMT  133 EG-DCLLLFLGHTPIFDVYRTLSHTEKWHSWLEHVDRFISPYHDNEDPEKEVKKIMERVGFSNIEVQCKTLFYVYDDLDVLKKSVAAINPFN--IPKDILEDFLEDYIDVVREMRLLDR-----CNNN------------
ApimeJHAMT  133 GG-KGLFMLLSWNDGFDVYKKLYANPRYRPYMQEPERFIPIFHECKDRRVNLRKILETTGFEILHCSEREKSYIYKNSEIMKKHIMAINPFISRIPNSLKKEFEDEITREIVNMKIQLLN----KDEN------------

SchgrJHAMT  278 DANSKKLTTRYSVLTVVAAKAAAQNGVRTVR-----------
DippuJHAMT  257 EEVT---TVSYDIIVAHIKKP---------------------
TricaJHAMT  256 NDNEEKIHVPYKLFVTFASKPV--------------------
DromeJHAMT  257 NEDQKFLSP-YKLVVAYARKTPEFVNNVFLEPTHQNLVKGIN
AedaeJHAMT  260 ENDYKFITP-YKLVVVYAVK----------------------
BommoJHAMT  253 VGESVSIKFNYKVISVYARKLCLSLM----------------
ApimeJHAMT  256 GEQEYNILDRYQIFVTYIRKPVC-------------------
```
