# Supplementary material for: Characterization of the Juvenile Hormone Pathway in the Viviparous Cockroach, Diploptera punctata
Source: PLoS One. 2015 Feb 23;10(2):e0117291. doi: 10.1371/journal.pone.0117291 (PMC4338245; doi:10.1371/journal.pone.0117291)
Supplement: S1 Table — Gene name abbreviations are shown in Fig. 1. (DOCX) [file pone.0117291.s003.docx]

**Table S1. (Degenerate) primer sequences for cloning of (partial) sequences for orthologous genes encoding JH biosynthesis enzymes in *D. punctata*. Gene name abbreviations were shown in Fig. 1.**

| **Gene name** | **F Primer(5'-3')** | **R Primer(5'-3')** | **Accession number (NCBI)** |
| --- | --- | --- | --- |
| ***Thiol*** | **GGDCARAATCCWGCDAGRCARGC** | **TTGHGCWGCRAANGCTTCRTT** | **KJ188021** |
| ***HMGS*** | **AGAGTGAACTGGAAGTGCA** | **TATGAAGCACGCACTCCTC** | **KJ188022** |
| ***HMGR*** | **AATGGTAGGGTGGCTGTTTCTA** | **GTATTTCATGATTTGCTGGGTTC** | **KJ188023** |
| ***MK*** | **CTGCCCCCGGTAAAGTTATC** | **GATGCTTGACTTCCTGCACC** | **KJ188024** |
| ***PMK*** | **ATGGAAGTAAACAAAATTTCAG** | **CACTCTGTTTCTGCATCATCTAC** | **KJ188025** |
| ***PPMD*** | **TGTTCAGAAAAYAATTTYCCNAC** | **TGATTNSWRTCYTTCATTGT** | **KJ188026** |
| ***IPPI*** | **CAYMGDGCVTTYAGTKTDTTY** | **TCDAWYTCRTGTTCKCCCCA** | **KJ188027** |
| ***FPPS*** | **TGGCGCGTAGGGTAGACAA** | **GATCCATAACATTCTGCAAACATTG** | **KJ188028** |
| ***FOLD*** | **ATGCAGCGTTGGACTGGA** | **TCCAGCATTGTTGATGAGAA** | **KJ188029** |
| ***JHAMT*** | **ATGCACAAAGCAGAACTGTATTC** | **TCAAGGCTTTTTAATATGAGCGACAAT** | **KJ188030** |
| ***CYP15A1*** | **ATGGTCATCGCTCTTATTGTCATC** | **ACCATTCATTTCCTTGGAATCAACT** | **AY509244** |
| ***Vg*** | **TGGAACRCDCTICTCTGYTGYCT** | **TTKAKSAYGTTRAYTTCCCAG** | **KJ188031** |
