# Supplementary material for: Characterization of the Juvenile Hormone Pathway in the Viviparous Cockroach, Diploptera punctata
Source: PLoS One. 2015 Feb 23;10(2):e0117291. doi: 10.1371/journal.pone.0117291 (PMC4338245; doi:10.1371/journal.pone.0117291)
Supplement: S2 Table — (DOCX) [file pone.0117291.s004.docx]

**Table S2. The Ct value of housekeeping genes (EF1α and Tubulin) in the CA and fat body sample.**

| **Tissue** |  |  |  |  |  | **Tissue** |  |  |  |  |  |
| --- | --- | --- | --- | --- | --- | --- | --- | --- | --- | --- | --- |
| **CA** | Gene | sample | Ct value | average |  | **Fat body** | Gene | sample | Ct value | average |  |
|  | EF1a | control 1 | 19.31 | 19.22 |  |  | EF1a | control 1 | 21.90 | 21.22 |  |
|  | EF1a | control 2 | 19.29 |  |  |  | EF1a | control 2 | 21.24 |  |  |
|  | EF1a | control 3 | 19.09 |  |  |  | EF1a | control 3 | 21.93 |  |  |
|  | EF1a | control 4 | 19.21 |  |  |  | EF1a | control 4 | 20.77 |  |  |
|  | EF1a | treated 1 | 19.18 | 19.46 |  |  | EF1a | control 5 | 20.25 |  |  |
|  | EF1a | treated 2 | 19.59 |  |  |  | EF1a | treated 1 | 21.93 | 21.54 |  |
|  | EF1a | treated 3 | 19.62 |  |  |  | EF1a | treated 2 | 22.30 |  |  |
|  |  |  |  |  |  |  | EF1a | treated 3 | 22.04 |  |  |
|  |  |  |  |  |  |  | EF1a | treated 4 | 21.20 |  |  |
|  |  |  |  |  |  |  | EF1a | treated 5 | 20.23 |  |  |
|  |  |  |  |  |  |  |  |  |  |  |  |
|  | Tubulin | control 1 | 21.68 | 21.68 |  |  | Tubulin | control 1 | 24.00 | 24.08 |  |
|  | Tubulin | control 2 | 21.60 |  |  |  | Tubulin | control 2 | 24.38 |  |  |
|  | Tubulin | control 3 | 21.58 |  |  |  | Tubulin | control 3 | 24.88 |  |  |
|  | Tubulin | control 4 | 21.87 |  |  |  | Tubulin | control 4 | 24.23 |  |  |
|  | Tubulin | treated 1 | 21.39 | 21.39 |  |  | Tubulin | control 5 | 22.91 |  |  |
|  | Tubulin | treated 2 | 21.73 |  |  |  | Tubulin | treated 1 | 24.91 | 24.16 |  |
|  | Tubulin | treated 3 | 21.73 |  |  |  | Tubulin | treated 2 | 25.37 |  |  |
|  |  |  |  |  |  |  | Tubulin | treated 3 | 25.39 |  |  |
|  |  |  |  |  |  |  | Tubulin | treated 4 | 22.55 |  |  |
|  |  |  |  |  |  |  | Tubulin | treated 5 | 22.59 |  |  |
